# Supplementary material for: Development and Properties of Fish Gelatin/Oxidized Starch Double Network Film Catalyzed by Thermal Treatment and Schiff’ Base Reaction
Source: Polymers (Basel). 2019 Dec 11;11(12):2065. doi: 10.3390/polym11122065 (PMC6960496; doi:10.3390/polym11122065)
Supplement: Supplementary file 1 [file polymers-11-02065-s001.zip › supplementary material.docx]

Fig.1 illustrated the mechanism of Schiff’ base reaction between gelation and oxidized starch.


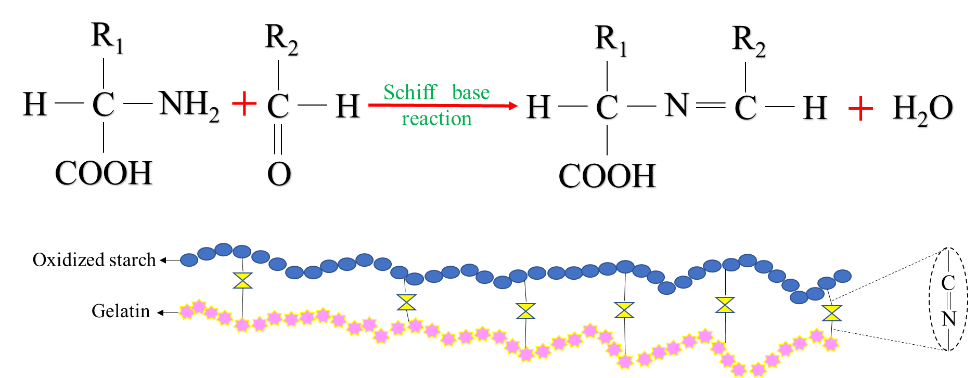


Fig.1 The Schiff’ reaction between gelatin and oxidized starch.

In the preliminary experiments, various FG concentration (ranging from 4% to 8%, w/v) was adopted to fabricate FG single gel. The thickness, moisture content and water vapor permeability were measured to present the properties of different FG concentration films (shown in Fig.2). The results showed that the thickness had no significant difference between 4%-8% FG films. While the MC of films showed a decrease trendency as the FG concentrantion increased, the 4% FG films had the highest MC (13.22%), followed by the 5% FG films (12.92%). What’s more, the 5% FG films showed the lowest WVP (2.79 gm^-1^d^-1^MPa^- 1^), which indicated the best barrier properties for moisture. In general, these results above indicated that the best properties of FG film were obtained at the concentration of 5% (w/v).

 Fig.2 The effect of FG concentration on the properties of films **(a)** thickness, **(b)** moisture content, **(c)** water vapor permeability
